# Cathepsins B, D, and G Are Expressed in Metastatic Head and Neck Cutaneous Squamous Cell Carcinoma

**DOI:** 10.3389/fonc.2021.690460

**Published:** 2021-09-21

**Authors:** Felix Humphries, Bridget Chang-McDonald, Josie Patel, Nicholas Bockett, Erin Paterson, Paul F. Davis, Swee T. Tan

**Affiliations:** ^1^Gillies McIndoe Research Institute, Wellington, New Zealand; ^2^Wellington Regional Plastic, Maxillofacial & Burns Unit, Hutt Hospital, Wellington, New Zealand; ^3^Department of Surgery, The Royal Melbourne Hospital, The University of Melbourne, Melbourne, VIC, Australia

**Keywords:** cutaneous, metastatic, squamous cell carcinoma, cathepsin, renin-angiotensin system, cancer stem cells, head and neck

## Abstract

**Aim:**

We have previously demonstrated the presence of two cancer stem cell (CSC) subpopulations within metastatic head and neck cutaneous squamous cell carcinoma (mHNcSCC) expressing components of the renin-angiotensin system (RAS), which promotes tumorigenesis. Cathepsins B, D and G are enzymes that constitute bypass loops for the RAS. This study investigated the expression and localization of cathepsins B, D, and G in relation to CSC subpopulations within mHNcSCC.

**Methods:**

Immunohistochemical staining was performed on mHNcSCC tissue samples from 20 patients to determine the expression and localization of cathepsins B, D, and G. Immunofluorescence staining was performed on two of these mHNcSCC tissue samples by co-staining of cathepsins B and D with OCT4 and SOX2, and cathepsin G with mast cell markers tryptase and chymase. Western blotting and quantitative reverse transcription polymerase chain reaction (RT-qPCR) were performed on five mHNcSCC samples and four mHNcSCC-derived primary cell lines, to determine protein and transcript expression of these three cathepsins, respectively. Enzyme activity assays were performed on mHNcSCC tissue samples to determine whether these cathepsins were active.

**Results:**

Immunohistochemical staining demonstrated the presence of cathepsins B, D and G in in all 20 mHNcSCC tissue samples. Immunofluorescence staining showed that cathepsins B and D were localized to the CSCs both within the tumor nests and peri-tumoral stroma (PTS) and cathepsin G was localized to the phenotypic mast cells within the PTS. Western blotting demonstrated protein expression of cathepsin B and D, and RT-qPCR demonstrated transcript expression of all three cathepsins. Enzyme activity assays showed that cathepsin B and D to be active.

**Conclusion:**

The presence of cathepsins B and D on the CSCs and cathepsin G on the phenotypic mast cells suggest the presence of bypass loops for the RAS which may be a potential novel therapeutic target for mHNcSCC.

## Introduction

Cutaneous squamous cell carcinoma (cSCC) is the second most common skin cancer, affecting most commonly the head and neck region (1). The incidence of cSCC is increasing in countries with a high solar ultraviolet index, in fair-skinned and aging populations (2). Cumulative exposure to ultraviolet solar radiation is the main risk factor for the development of cSCC, leading to increased incidence with age (3), at an average age of onset of mid-60s (4), more commonly in men than women (5).

cSCC is the most common cancer capable of undergoing metastasis (6). 14% of head and neck cSCC (HNcSCC) are considered high-risk lesions (4) and up to 5% develop metastasis to the regional nodes (7). The risk of recurrence and metastasis of cSCC is increased with tumor diameter of >2cm, tumor depth of >2mm, invasion beyond the subcutaneous fat, the presence of perineural invasion, and poor differentiation (8). Metastatic HNcSCC (mHNcSCC) is an aggressive disease with a 5-year survival of 34-44% despite intensive treatment that typically involves surgery and adjuvant radiotherapy (9, 10). This poor prognosis has been attributed to the presence of cancer stem cells (CSCs) (11).

CSCs, the proposed origin of cancer, sustain cancer, resist radiotherapy and chemotherapy and are responsible for cancer metastasis and recurrence (12, 13). CSCs are highly tumorigenic and possess embryonic stem cell (ESC) characteristics, including pluripotency and self-renewal capacity (12, 14). CSC subpopulations have been identified in many cancer types (15–23) including primary HNcSCC (24) and mHNcSCC (25) that express transcription factors OCT4, SOX2, NANOG, KLF4 and c-MYC that are involved in generation of induced pluripotent stem cells (iPSCs) (26–28). We have previously identified two CSC subpopulations within mHNcSCC: an OCT4+/SOX2+/NANOG+/KLF4+/c-MYC+ subpopulation within the tumor nests (TNs) and an OCT4+/SOX2+/NANOG-/KLF4+/c-MYC+ subpopulation within the peritumoral stroma (PTS) (25).

Cathepsins are a class of globular proteases (29) and their proteolytic activity underscore certain hallmarks of cancer (30). The levels of cathepsins are normally regulated in a well-coordinated manner, and their activity is upregulated in cancer (31). Cathepsins and chymase promote metastasis – a multi-step process, by suppressing inhibitors of active pro-oncogenic proteases, leading to degradation of extra-cellular matrix (ECM) and basement membranes, and promoting angiogenesis (31–33). Cathepsins also prevent cell death through modulation of the lysosomal apoptotic pathways and promote cancer cell invasion through upregulation of cell adhesion and migration (30). Cathepsins B and D are expressed by CSCs in glioblastoma (34), oral tongue SCC (OTSCC) (35), metastatic colon adenocarcinoma to the liver (36) and primary HNcSCC (37).

Cathepsin B overexpression has been associated with cancer invasion and metastasis in many cancer types (31, 38, 39). It enhances the activity of matrix metalloproteinases (MMPs) through destruction of their inhibitors, promoting ECM degradation, and angiogenesis (40). Cathepsin D is associated with poor prognosis in many cancer types (41–43) including esophageal SCC (44). It increases the incidence of metastasis in breast cancer by promoting cell growth and decreased contact inhibition (45). Cathepsin G is a serine protease involved in degradation of extracellular components, antigen presentation, and leukemogenesis, and is associated with aggressive phenotypes in acute lymphoblastic leukemia and breast cancer (46–48).

Components of the renin-angiotensin system (RAS) are expressed by CSCs in many cancer types (49–55) including primary HNcSCC (56) and mHNcSCC (57). RAS signaling affects the hallmarks of cancer including cell proliferation, migration, invasion and metastasis (58–60). Targeting RAS with RAS inhibitors (RASis) may reduce tumor growth and improve delivery and efficacy of anti-cancer drugs (61). Cathepsin B, D, and G and chymase constitute bypass loops of the RAS (62), reducing the effectiveness of RAS blockade by RASis.

This study investigated the expression of cathepsins B, D and G and their localization in relation to the CSC subpopulations we have recently identified in mHNcSCC (25).

## Materials and Methods

### mHNcSCC Tissue Samples

mHNcSCC tissue samples from one female and 19 male patients aged 51-86 (mean, 77) years (**Supplementary Table 1**) including those used in our previous studies (25, 57), were sourced from the Gillies McIndoe Research Institute Tissue Bank. This study was approved by the Central Health and Disability Ethics Committee (Ref. 12/CEN/74AM05), with written informed consent from all participants.

### mHNcSCC-Derived Primary Cell Lines

Primary cell lines were generated from four available freshly excised mHNcSCC tissue samples of the original cohort of 20 patients. Samples were cut into small pieces and incubated between layers of Matrigel (cat#354234, Corning Life Sciences, Tewksbury, USA) in 24-well plates with a culture media containing Dulbecco’s Modified Eagle Medium (DMEM) with Glutamax™ (cat#15140122, Gibco, Rockford, IL, USA) supplemented with 2% penicillin-streptomycin (cat#15140122, Gibco) and 0.2% gentamycin/amphotericin B (cat#R01510, Gibco). Once sufficient cell growth was achieved to support transfer to a monolayer culture, cells were extracted by dissolving the Matrigel with Dispase (cat#354235, Corning Life Sciences) and transferred to an adherent culture flask with media consisting of DMEM (1X) (Gibco) with Glutamax™ supplemented with 10% fetal bovine serum (cat#10091148, Gibco), 5% mTeSR™1 Complete Medium (cat#85850, STEMCELL Technologies, Vancouver, BC, Canada), 1% penicillin-streptomycin and 0.2% gentamycin-amphotericin (Gibco) in a humidified incubator at 37°C and 5% CO_2_. Cells were expanded in culture and harvested between passages 4 and 8.

### Histochemical and Immunohistochemical Staining

Hematoxylin and eosin (H&E) staining was performed on 4 μm-thick formalin-fixed paraffin-embedded sections of mHNcSCC tissue samples from 20 patients. The presence of mHNCSCC was confirmed on H&E stained slides by an anatomical pathologist. Immunohistochemical staining of tissue sections for cathepsin B (1:200, cat#NBP119797, Abcam), cathepsin D (1:2000, cat#AB75852, Abcam) and cathepsin G (1:100, cat#NBP233498, Novus Biological) was performed on the Leica BOND™ RX auto-stainer (Leica, Nussloch, Germany) using the BOND Polymer Refine Detection (cat#9800, Leica), using 3,3’-diaminobenzidine as the chromogen. Immunohistochemical-stained slides were mounted in Dako Mounting Medium (cat#CS703, Dako, Glostrup, Denmark) and coverslipped using a Dako coverslipper.

Human tissues used for positive controls were placenta for cathepsin B, breast cancer for cathepsin D, tonsil for cathepsin G. Negative controls were mHNcSCC sections run with mouse (ready-to-use; cat#DK1594, Dako) and rabbit (ready-to-use; cat#DK1594, Dako) primary antibodies.

### Immunofluorescence Staining

To confirm co-expression of two proteins, immunofluorescence staining was performed on two of the 20 mHNcSCC tissue samples, using the same primary antibodies with the same concentrations as used for immunohistochemical staining, and co-staining with OCT4 (1:30; cat#309M-16, Abcam) or SOX2 (1:500; PA1-094, Thermo Fisher Scientific) which was used as a surrogate marker for the CSC subpopulations we have recently identified in mHNcSCC (25), and mast cell markers tryptase (1:300; cat#nCL-MCTRYP-428, Leica) and chymase (1:1500, cat#PA528317, Invitrogen). Because of species compatibility, to demonstrate the co-localization of cathepsin B with SOX2 or cathepsin D, a different cathepsin B primary antibody (1:200; cat#ab58802, Abcam) was used for some of the immunofluorescence work. Appropriate secondary antibodies and amplification kits were used for immunofluorescence detection; Alexa Fluor anti-mouse 488 (1:500; cat#A-21202, Life Technologies, Carlsbad, CA, USA), Alexa Fluor anti-rabbit 594 (1:500; cat#A-21207, Life Technologies), VectaFluor Excel anti-mouse 488 (ready-to-use; cat#DK-2488, Vector Laboratories, Burlingame, CA, USA), and VectaFluor Excel anti-rabbit 594 (ready-to-use; cat#DK-1594, Vector Laboratories). All antibodies were diluted in BOND primary antibody diluent (cat#AR9352, Leica). Slides were mounted using Vectashield hardset medium with 4’,6-diamidino-2-phenylindole (cat#H-1500, Vector Laboratories).

Negative controls for immunofluorescence staining were performed on mHNcSCC sections using primary isotype mouse (ready-to-use; cat#DK1594, Dako) and rabbit (ready-to-use; cat#DK1594, Dako) isotype controls. All immunofluorescence staining was performed on the Leica BOND™ RX auto-stainer.

### Image Analysis

Immunohistochemical-stained slides were viewed, and the images were captured on the Olympus BX53 light microscope fitted with an Olympus SC100 camera (Olympus, Tokyo, Japan), and processed with cellSens 2.0 software (Olympus). Immunofluorescence-stained slides were viewed and imaged with an Olympus FV1200 biological confocal laser-scanning microscope and processed with cellSens Dimension 1.17 software (Olympus).

### Reverse Transcription Quantitative Polymerase Chain Reaction

Total RNA was isolated from five of the six available snap-frozen mHNcSCC tissue samples and four available mHNcSCC-derived primary cell lines from the original cohort of 20 patients. From frozen cell pellets of 5x10^5^ viable cells, RNA was extracted using the RNeasy Micro kit protocol (cat#74004, Qiagen). From the tissue samples, approximately 20mg was homogenized using the Omno Tissue Homogenizer (Omni TH, Omni International, Kennesaw, GA, USA) in 350 µl of RLT lysis buffer before continuing with the RNeasy Mini kit protocol (cat#74104, Qiagen). An on-column DNase digest (cat#79254, Qiagen) step was included to remove potentially contaminating genomic DNA. Total RNA quantity was determined using a NanoDrop 2000 Spectrophotometer (Thermo Fisher Scientific). Transcriptional expression was analyzed in triplicate using the Rotor-Gene Q (Qiagen), Rotor-Gene Multiplex RT-PCR Kit (cat#204974, Qiagen) and TaqMan Gene Expression Assay primer probes (cat#4331182, Thermo Fisher Scientific) on 40 ng of RNA. The TaqMan primer probes used were; cathepsin B (Hs00157194_m1), cathepsin D (Hs00157205_m1), cathepsin G (Hs01113415_g1). Gene expression was normalized to the reference genes GAPDH (Hs99999905_m1) and PUM1 (Hs00160598_m1; cat#4331182, Thermo Fisher Scientific). Universal human reference RNA (UHR; cat#CLT636690, Takara, Shiga, Japan), total RNA extracted from a range of healthy adult human tissues, was used as the calibrator for the 2^ΔΔCt^ analysis. Human tonsil tissue was included as a positive control, and nuclease free water was added for the no template control. A no reverse transcriptase control was included for those assays which may detect gDNA. End-point amplification products were checked for the presence of the correctly sized band by gel electrophoresis on 2% agarose gel electrophoresis (cat#G402002, Thermo Fisher Scientific) and imaged using the ChemiDoc MP (Bio-Rad, Hercules, CA, USA). Graphs were generated using GraphPad Prism (v8.0.2, San Diego, CA, USA) and results expressed as fold-change relative to UHR. A fold-change cut off was set at 2.0 for up-regulated, and 0.5 for down-regulated genes.

### Western Blotting

Total protein was extracted from the same five snap-frozen mHNcSCC tissue samples and four mHNcSCC-derived primary cell lines used for RT-qPCR. Cell pellets were lysed in ice-cold Radioimmunoprecipitation assay buffer (cat#89900, Pierce Biotechnology, Rockford, IL, USA) supplemented with a protease and phosphatase inhibitor cocktail (cat#78440, Pierce Biotechnology). Protein was quantified using a BCA assay (cat#23227, Pierce Biotechnology) and subsequently diluted in an equal volume of 2x LDS (cat#B0007, Invitrogen). 20µg of total protein was separated by SDS-PAGE on 4-12% Bis-Tris gels (cat#NW04122BOX, Invitrogen) in MES SDS running buffer (cat#B0002, Invitrogen) and transferred to a PVDF membrane (cat#IB24001, Invitrogen) using an iBlot 2 (cat#IB21001, Thermo Fisher Scientific). Protein was detected on the iBind Flex (cat#SLF2000, Thermo Fisher Scientific) using primary antibodies for cathepsin B (1:1000; cat#Ab58802, Abcam) and cathepsin D (1:1000; cat#Ab6313, Abcam). Secondary antibodies used were goat anti-rabbit HRP (1:1000; cat#ab6721 Abcam) for cathepsins D, goat anti-mouse HRP (1:1000; cat#ab6789, Abcam) for cathepsin B, and goat anti-mouse Alexa488 (1:1000; cat#A21202, Life Technologies, Carlsbad, CA, USA) for the loading control a-tubulin (1:2000; cat#62204, Thermo Fisher Scientific).

To visualize HRP protein bands, Clarity Western ECL substrate (cat#1705061, Bio-Rad) was used with the ChemiDoc MP Imaging System (Bio-Rad) and Image Lab 6.0 software (Bio-Rad) to analyze protein bands.

### Enzymatic Activity Assays

Enzymatic activity of cathepsins B and D was determined in snap-frozen mHNcSCC tissue samples from the same patients whose samples were analyzed by RT-qPCR, using enzymatic activity assay kits for cathepsin B (cat#ab65300; Abcam) and cathepsin D (cat#ab65302; Abcam), according to the manufacturer’s protocol. This was performed on six tissue samples for cathepsin B, and three tissues samples for cathepsin D because three of the six samples (samples 1, 3 and 6) were exhausted. Fluorescence was measured in a Nunc™ F96 MicroWell™ black polystyrene plate (cat#136101, Thermo Fisher Scientific) using the Varioskan Flash plate reader (cat#MIB5250030, Thermo Fisher Scientific). Tonsil and denatured tonsil tissue lysates were used as appropriate positive and negative controls, respectively. All experiments were performed in duplicate with averages taken.

## Results

### Cathepsins B, D, and G Were Expressed in mHNcSCC Tissue Samples

**Figure 1:**
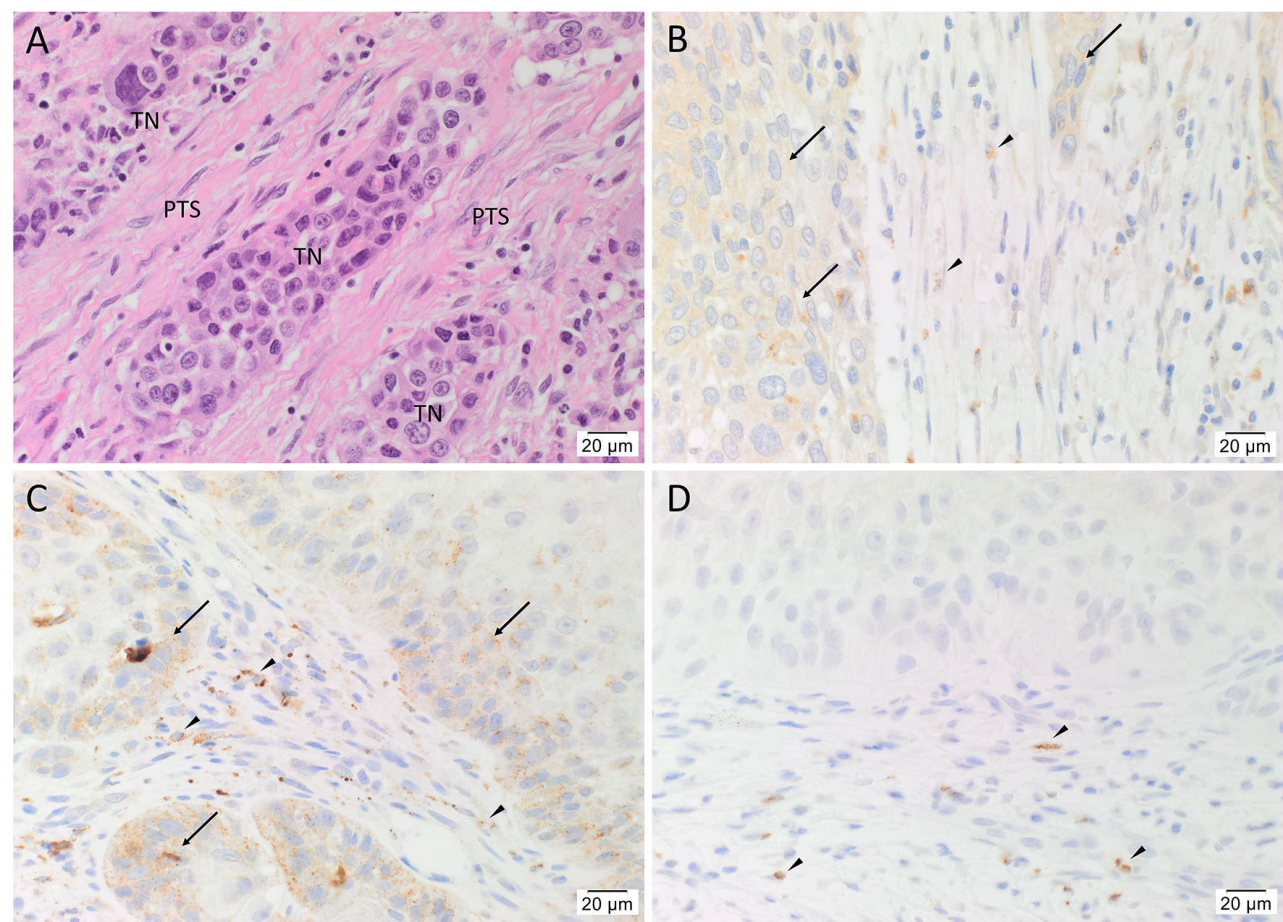
Representative hematoxylin and eosin **(A)** and immunohistochemical-stained **(B–D)** images of metastatic head and neck cutaneous squamous cell carcinoma (mHNcSCC) tissue samples, demonstrating tumor nests (TNs) surrounded by the peritumoral stroma (PTS) **(A)**. Cathepsin B (**B**, brown) and cathepsin D (**C**, brown) were expressed on the cytoplasm of cells within the TNs (arrows), and occasional cells within the PTS (arrowheads). Cathepsin G (**D**, brown) was expressed by few cells within the PTS (arrowheads). Nuclei were counter-stained with hematoxylin. Original magnification: 400x. Scale bars: 20µm. n = 20.

H&E staining confirmed the diagnosis of mHNcSCC in all 20 tissue samples which was organized into TNs surrounded by the PTS (**Figure 1A**). Immunohistochemical staining demonstrated moderate cytoplasmic expression of cathepsin B (**Figure 1B**, arrows) and cathepsin D (**Figure 1C**, arrows) by cells within the TNs, and to a lesser extent, cells within the PTS (**Figures 1A, B**, arrowheads) in all 20 mHNcSCC tissue samples. Cytoplasmic expression of cathepsin G was present in cells within the PTS (arrowheads) with no staining of the TNs (**Figure 1D**). The staining patterns of cathepsins B, D and G of all 20 mHNcSCC tissue samples are shown in **Supplementary Table 2**.

Human tissues used for positive controls demonstrated the expected staining patterns for cathepsin B on placenta (**Supplementary Figure 1A**), cathepsin D on breast carcinoma (**Supplementary Figure 1B**), cathepsin G (**Supplementary Figure 1C**) on tonsil. Specificity of the secondary antibodies was confirmed on sections of mHNcSCC tissue samples using a matched isotype control for both mouse and rabbit primary antibodies (**Supplementary Figure 1D**).

### Cathepsins B and D Were Expressed by CSCs and Cathepsin G by Mast Cells in mHNcSCC Tissue Samples

**Figure 2:**
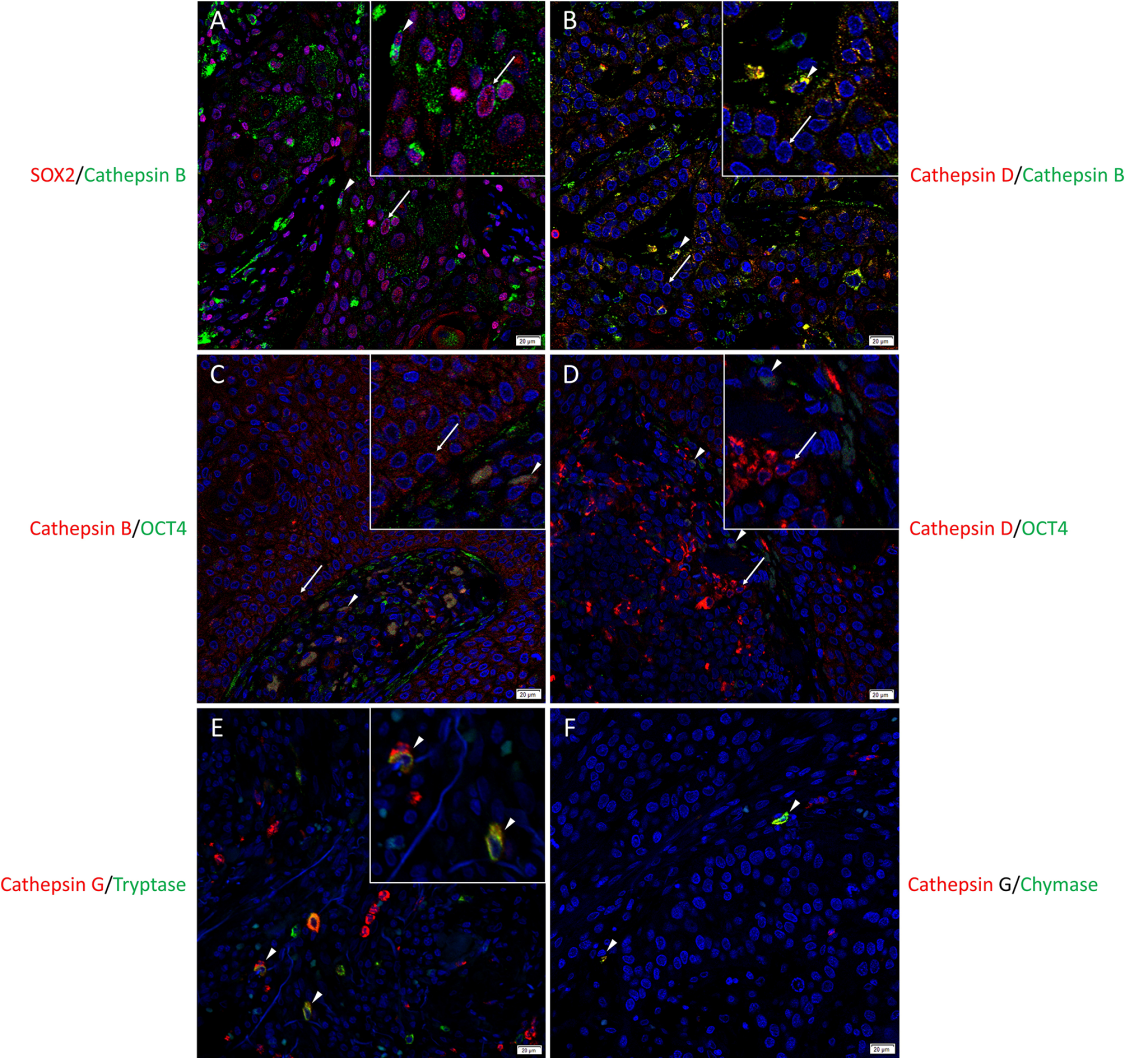
Representative immunofluorescence-stained sections of a metastatic cutaneous head and neck squamous cell carcinoma tissue sample demonstrating the co-expression of cathepsins B and D with embryonic stem cell markers. Cathepsin B (**A**, green) was expressed by the SOX2+ (**A**, red) CSCs within the tumor nests (TNs) and to a lesser extent those within the peritumoral stroma (PTS). Cells that expressed cathepsin B (**B**, green) also expressed cathepsin D (**B**, red**)**. Cathepsin B (**C**, red) and cathepsin D (**D**, red) were expressed by OCT4+ (**D, E**, green) CSCs within the PTS. Cathepsin G (**E, F**, red) were expressed by cells within the PTS, majority of which expressed tryptase (**E**, green) and few expressed chymase (**F**, green). All slides were counter-stained with 4′,6-diamidino-2-phenylindole (**A–F**, blue). Original magnification 400x. Scale bars: 20µm. n = 2. The inserts show enlarged views of the corresponding images.

To localize the cathepsins in relation to the CSC sub-populations we have previously identified (25), immunofluorescence dual-staining was performed on two representative mHNcSCC samples from the original cohort of 20 patients. This demonstrated expression of cathepsin B (**Figure 2A**, green) by the SOX2+ (**Figure 2A**, red) CSCs within the TNs (arrows) and the PTS (arrowheads). Most of the cathepsin B+ (**Figure 2B**, green) cells within the TNs (arrows) and the PTS (arrowheads) expressed cathepsin D (**Figure 2B**, red). Cathepsin B (**Figure 2C**, red) and cathepsin D (**Figure 2D**, red) were expressed by the OCT4+ (**Figures 2C, D**, green) CSCs within the PTS (arrowheads) and some OCT4+ (**Figures 2C, D**, green) CSCs within the TNs (arrows). Cathepsin G (**Figure 2E**, red) was expressed by cells within the PTS, majority of which expressed tryptase (**Figure 2F**, green) and some of which expressed chymase (**Figure 2F**, green), markers of mast cells (63). Figure inserts have been provided to show enlarged views of the corresponding images.

Individual stains demonstrated in **Figure 2** are presented in **Supplementary Figures 2A–N**. Minimal staining was present on the negative control (**Supplementary Figure 2M**) confirming the specificity of the primary antibodies used.

### Cathepsins B, D, and G Transcripts Were Expressed in mHNcSCC Tissue Samples and mHNcSCC-Derived Primary Cell Lines

**Figure 3:**
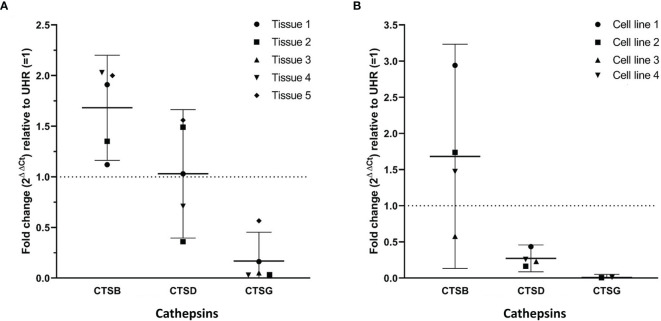
Graphs showing 2^ΔΔCt^ values of RT-qPCR runs performed on five snap-frozen metastatic cutaneous head and neck squamous cell carcinoma (mHNcSCC) tissue samples **(A)** and four mHNcSCC-derived primary cell lines **(B)**, amplifying transcripts for cathepsin B, cathepsin D and cathepsin G. ΔΔCt was calculated by normalizing CT values of cathepsins D, B and G to that of the reference genes GAPDH and PUM1, and then expressing this relative to the ΔCt of healthy UHR. Error bars: 95% confidence interval.

RT-qPCR performed on five mHNcSCC tissue samples (no sufficient RNA was extracted from sample 6) (**Figure 3A**) and four mHNcSCC-derived primary cell lines (**Figure 3B**) confirmed expression of cathepsins B and D, while cathepsin G was detected in all five tissue samples and two of four cell lines. There was a biologically insignificant increase in mean cathepsin B expression in both the tissue samples and the cell lines, relative to UHR. Cathepsin D expression in the mHNcSCC tissue samples showed no significant change but was downregulated in the cell lines, relative to UHR. Cathepsin G expression was downregulated in four of the five mHNcSCC tissue samples and two of the four cell lines, relative to UHR.

Specific amplification of the products was demonstrated by electrophoresis of qPCR products on 2% agarose gels. The expected size amplicons were observed, and no products were observed in the no template control reactions (**Supplementary Figure 3**).

### Cathepsins B and D Proteins Were Present in mHNcSCC Tissue Samples and mHNcSCC-Derived Primary Cell Lines

**Figure 4:**
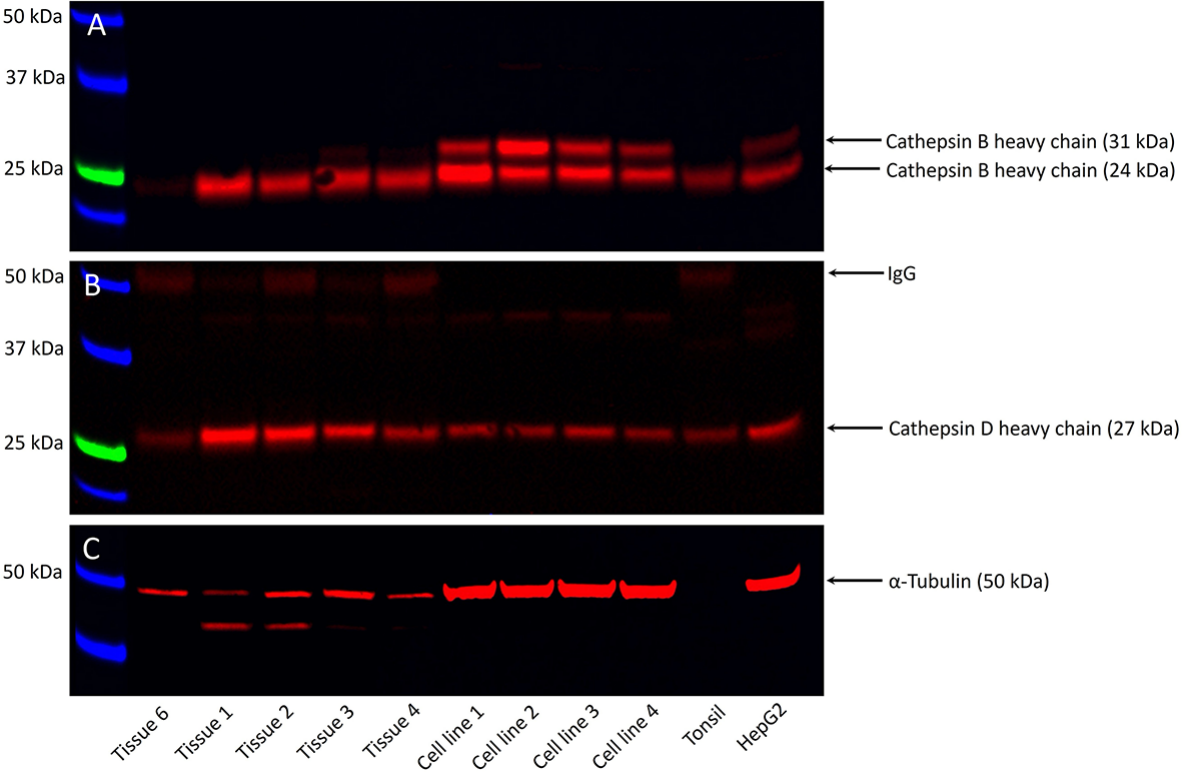
Representative Western blot images of total protein extracted from five metastatic cutaneous head and neck squamous cell carcinoma (mHNcSCC) tissue samples and four mHNcSCC-derived primary cell lines, demonstrating the presence of cathepsin B in four out of the five tissue samples and all four cell lines **(A)**, and cathepsin D in all five tissue samples and all four cell lines **(B)**. Relatively equal amounts of proteins were loaded into each lane, as confirmed by α-tubulin **(C)**. The molecular weight ladder (kDa) is labelled for each blot.

WB analysis showed the presence of bands at the expected molecular weights for cathepsins B in four of the five, and cathepsin D in all five, snap-frozen mHNcSCC tissue samples, and all four mHNcSCC-derived primary cell lines (**Figure 4**) included in WB analysis. Moderate expression of cathepsin B was detected at the appropriate molecular weight of 24 kDa in four out of the five tissue samples. There were two bands present in all four mHNcSCC-derived primary cell lines, at the appropriate molecular weights of 24 kDa and 29 kDa. The 24 kDA band is representative of the carbohydrate-free heavy chain form, with the 29 kDa band representing the mature single-chain enzyme (64). Cathepsin D was detected at the corresponding molecular weight of 27 kDa in all five tissue samples, and in all four of the mHNcSCC-derived cell lines. This 27 kDa subunit is representative of the cathepsin D heavy chain (65). Approximately equal amounts of proteins were loaded into each lane, as confirmed by α-tubulin staining, although tissue samples had lower α-tubulin levels. Cathepsin G was not investigated by WB as it was expressed by scattered cells in the mHNcSCC tissues.

### Cathepsins B and D in mHNcSCC Tissue Samples Were Active

**Figure 5:**
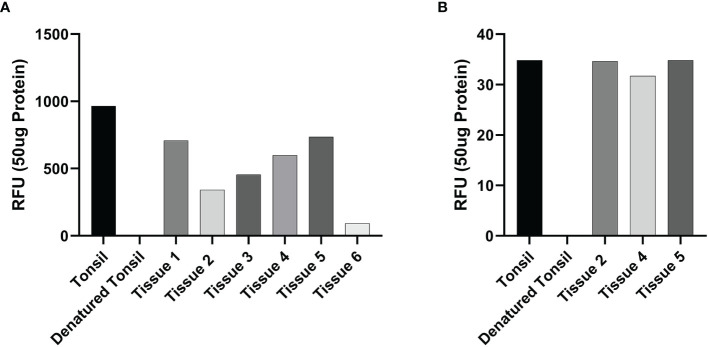
Averages for the enzymatic activity assays of metastatic cutaneous head and neck squamous cell carcinoma (mHNcSCC) tissue samples for cathepsin B and cathepsin D This showed cathepsin B **(A)** in all six tissue samples and cathepsin D **(B)** in all 3 samples were active, relative to the positive (tonsillar) and negative (denatured tonsillar) control tissues. Their activity is expressed as relative fluorescent unit (RFU).

Enzymatic activity assays demonstrated enzymatic activity of cathepsin B (**Figure 5A**) and cathepsin D (**Figure 5B**), on all six and three mHNcSCC tissue samples, respectively, relative to that of the positive and negative control tissues.

## Discussion

The incidence of cSCC is increasing in countries with a high solar ultraviolet index in fair-skinned and aging populations (2), most commonly in the head and neck region (1). Up to 5% of HNcSCC develop metastasis to the regional nodes (7) with a 34-48% 5-year overall survival rate despite surgery and adjuvant radiotherapy (9, 10). Systemic therapies including chemotherapy, 13-cis-retinoic acid, interferon-α2a, and targeted therapy have been used for advanced diseased with modest efficacy (66). The poor survival of mHNcSCC has been attributed to the presence of CSCs which possess self-renewal ability that contribute to treatment resistance, cancer metastasis and recurrence (67). CSCs have been proposed to invade surrounding tissue and metastasize by acquiring an epithelial-to-mesenchymal transition phenotype (68).

In this study, immunohistochemical staining demonstrated the presence of cathepsins B and D in the TNs and the PTS, while cathepsin G was localized to cells within the PTS, of mHNcSCC tissues. This finding is supported by results of RT-qPCR analysis which demonstrated transcript expression of cathepsins B, D and G in mHNcSCC tissue samples. Transcript expression of cathepsins B and D, but not cathepsin G, in mHNcSCC-derived primary cell lines, is consistent with the widespread expression of cathepsins B and D by cells within the TNs and PTS, whereas cathepsin G was expressed by few cells within the PTS that may not have survived the culturing process. The presence of cathepsins B and D proteins in mHNcSCC tissues and primary cell lines was confirmed by WB, with the bands representing the mature and heavy-chain forms of cathepsins B and D. Enzyme activity assays demonstrated that these cathepsins in the mHNcSCC tissue samples were active.

We have previously demonstrated expression of cathepsins B, and D by CSCs in a number of cancer types (34–36) including primary HNcSCC (37). In this study, immunofluorescence staining demonstrated localization of cathepsins B and D to the CSCs within both the TNs and the PTS, and cathepsin G on the phenotypic mast cells within the PTS, similar to OTSCC (35). The co-localization of cathepsin B and cathepsin D has been previously demonstrated in cSCC, with cooperation between the two enzymes suggested to play a role in tumor invasion and metastasis (69).

Cathepsins B, D and G exert different functions by activating and inhibiting a vast network of proteolytic interactions that contribute to cancer progression and metastasis (30, 32), by interacting with signaling pathways involving chemokines, cytokines and kinases (30). This process results in promoting certain hallmarks of cancer, such as tumor cell invasion of the stroma, angiogenesis and metastasis, and also cell proliferation, protein and ECM degradation, and activation of other proteases to cleave pro-peptides (30, 32). Cysteine cathepsins are usually located in intracellular compartments in normal cells, however, they can be translocated to the cell surface and possibly secreted in tumors (70). While cathepsins B and D act mostly extracellularly in their promotion of cancer progression, their intracellular activity may contribute to tumorigenesis, as inhibition of intracellular cathepsins has been shown to result in increased tumor cell death and a reduced tumor size in several murine models (30, 71–73).

Cathepsin B stains more intensely in SCC tumor cells than in the epidermis of normal skin (69). Overexpression of cathepsin B transcripts and proteins and elevated cathepsin B activity are present in the invasive edge of various human cancers (32). Raised levels of cathepsin B have been found in metastatic malignant melanoma and lung cancer tissues of patients with hematogenous and intrapulmonary metastasis, and in tumor infiltrated lymph nodes (32). Anicin et al. (74) demonstrate that laryngeal SCC patients who show protein expression of the cathepsin inhibitor stefin A have a better survival outcome than those without expression of stefan A. Similarly, Li et al. (75) show that overexpression of stefin A in esophageal SCC is associated with improved survival.

Vigneswaran et al. (76) show a strong relationship between the expression levels of cathepsins B and D and the rate of local invasion and metastases in oral cavity SCC patients, suggesting that they are useful prognostic markers and potential therapeutic targets (77). Patients with metastatic laryngeal SCC that demonstrate the presence of cathepsin D positive tumor cells are at a higher risk of cancer relapse than those without these tumor cells (78).

In this study, immunofluorescence staining demonstrated expression of cathepsin G by cells within the PTS, many of them expressed tryptase and some expressing chymase, markers of mast cells (63). Mast cells contribute to tumorigenesis of SCC through multiple functions including immunosuppression, ECM degradation, mitogenesis, and angiogenesis (79). Mast cells contribute to angiogenesis by releasing tryptase and chymase that help initiate matrix degradation and turnover (33, 80, 81). Chymase can also activate latent MMPs which degrade components of the epithelial basement membranes and ECM, responses that are essential for tumor invasion and metastasis (82).

CSCs have been demonstrated in many cancer types (15–23) including primary HNcSCC (24) and mHNcSCC (25). Components of the RAS are expressed by CSCs in many cancer types (49–55) including primary HNcSCC (56) and mHNcSCC (57). RAS signaling has been shown to affect cell proliferation, migration, invasion, metastasis, apoptosis, angiogenesis, cancer associated inflammation, immunomodulation and tumor fibrosis/desmoplasia (58, 59). RAS signaling increases cell proliferation in cancer by directly affecting tumor and stromal cells and indirectly by modulating the growth of vascular cells during angiogenesis (58). A reduction in tumor growth have been found following RAS blockade in experimental models (59). We have shown expression of components of the RAS: pro-renin receptor, angiotensin-converting enzyme (ACE), angiotensin II (ATII) receptor 1 (AT_1_R) and ATII receptor 2 (AT_2_R) by CSCs in mHNcSCC (57).

Classically a hormone system that regulates blood pressure and fluid and electrolyte balance, RAS signaling plays a role in tumorigenicity, including cell proliferation, migration, angiogenesis and metastasis (58, 59, 61). Components of the RAS are expressed in a number of cell types such as endothelial cells, fibroblasts, monocytes, macrophages, neutrophils, dendritic cells, and T cells within the tumor microenvironment (58) which regulates CSCs (83–85).

The RAS also promotes cancer-related inflammation and infiltration of tumor promoting immune cells (61). ATII/AT_1_R signaling also promotes vascular endothelial growth factor-mediated angiogenesis in solid tumors (61). Targeting ATll/AT_2_R signaling with RASis may potentially reduce tumor desmoplasia, decrease solid stress, increase tumor perfusion, reduce hypoxia, enhance T cell infiltration and anti-tumor immunity, and improve delivery and efficacy of anti-cancer drugs (61). However, bypass loops consisting of proteases such as cathepsins B, D and G (62) exist causing redundancy of the RAS, rendering RAS inhibition less effective. Cathepsin B is able to catalyze the conversion of pro-renin into active renin (86). Cathepsin G can generate ATII from angiotensin I (ATI) and directly from angiotensinogen; and cathepsin D converts angiotensinogen to ATI (32, 34, 87). Targeting these cathepsins alongside RASis may potentially reduce the availability of ATII and reduce its pro-cancer effects. This novel therapeutic approach of combining RAS and cathepsin inhibitors may effectively target CSCs in the treatment of cancer (62, 88) including mHNcSCC.

## Conclusion

This study demonstrates expression of cathepsins B and D by CSCs, and cathepsin G by mast cells within the mHNcSCC. These findings may open up avenues for novel therapeutic targeting of CSCs in the treatment of this aggressive cancer.

### Limitations

This study includes a relatively small sample size, especially for RT-qPCR, WB and enzyme activity assays. However, novel findings from this study provide the foundation for future larger studies. Further *in vitro* and *in vivo* experiments are required to confirm the functional role of cathepsins B, D and G in the regulation of CSCs in mHNcSCC.

## Data Availability Statement

The raw data supporting the conclusions of this article will be made available by the authors, without undue reservation.

## Ethics Statement

This study was approved by the Central Health and Disability Ethics Committee (Ref. 12/CEN/74AM05). Written consent was obtained from all participants who provided their written informed consent to participate in this study.

## Author Contributions

ST conceptualized and designed the study. NB performed confocal microscopy and western blotting. NB, FH, PD and ST interpreted the immunofluorescence and western blotting results. JP performed RT-qPCR. JP, FH, ST interpreted the RT-qPCR results. EP performed cell culture experiments and the enzymatic activity assays and interpreted the results. FH, BC-M, PD and ST interpreted the immunohistochemical staining results. ST supervised and administered the project. FH and ST drafted the manuscript. NB, JP, EP, BC-M, PD, and ST edited the manuscript. All authors contributed to the article and approved the submitted version.

## Funding

This research was funded by the Head and Neck Cancer Foundation Aotearoa and Gillies McIndoe Research Institute internal fund. FH was supported by a Deane Endowment Trust Summer Student Scholarship.

## Conflict of Interest

PD and ST are inventors of the patents Cancer Diagnosis and Therapy (PCT/NZ2015/050108), Cancer Therapeutic (PCT/NZ2018/050006), Novel Pharmaceutical Compositions for Cancer Therapy (US/62/711709) and Cancer diagnosis and therapy (United States Patent No. 10281472).

The remaining authors declare that the research was conducted in the absence of any commercial or financial relationships that could be construed as a potential conflict of interest.

## Publisher’s Note

All claims expressed in this article are solely those of the authors and do not necessarily represent those of their affiliated organizations, or those of the publisher, the editors and the reviewers. Any product that may be evaluated in this article, or claim that may be made by its manufacturer, is not guaranteed or endorsed by the publisher.
