# Supplementary material for: Cathepsins B, D, and G Are Expressed in Metastatic Head and Neck Cutaneous Squamous Cell Carcinoma
Source: Front Oncol. 2021 Sep 21;11:690460. doi: 10.3389/fonc.2021.690460 (PMC8491843; doi:10.3389/fonc.2021.690460)
Supplement: Supplementary file 1 [file DataSheet_1.docx]

**Cathepsins B, D and G Are Expressed in Metastatic Head and Neck Cutaneous Squamous Cell Carcinoma**

**Supplementary Materials**

**SupplementaryTable 1.** Patient demographics and site of nodal metastatic head and neck cutaneous squamous cell carcinoma

| Patient | Sex | Age (years) | Site of Nodal Metastasis |
| --- | --- | --- | --- |
| 1 | M | 58 | Neck |
| 2 | M | 76 | Parotid and neck |
| 3 | M | 86 | Parotid |
| 4 | M | 83 | Parotid and neck |
| 5 | M | 85 | Parotid |
| 6 | M | 81 | Parotid and neck |
| 7 | M | 86 | Neck |
| 8 | M | 80 | Parotid |
| 9 | M | 51 | Neck |
| 10 | M | 75 | Neck |
| 11 | M | 74 | Neck |
| 12 | M | 74 | Neck |
| 13 | M | 73 | Parotid |
| 14 | M | 83 | Neck |
| 15 | M | 77 | Parotid |
| 16 | M | 85 | Parotid |
| 17 | M | 77 | Parotid and neck |
| 18 | M | 81 | Parotid and neck |
| 19 | M | 77 | Parotid |
| 20 | F | 86 | Parotid |

M, male; F, female

**Supplementary Table 2.** Immunohistochemical staining patterns of cathepsins B, D and G in metastatic head and neck cutaneous squamous cell carcinoma in 20 patients

| Patient | Cathepsin B | | | Cathepsin D | | | Cathepsin G | | |
| --- | --- | --- | --- | --- | --- | --- | --- | --- | --- |
|  | PTS | TNs | PTS | | TNs | PTS | | TNs |  |
| 1 | + | ++ | + | | ++ | - | | - |  |
| 2 | ++ | + | ++ | | + | + | | + |  |
| 3 | ++ | ++ | + | | +++ | - | | - |  |
| 4 | ++ | + | + | | ++ | + | | - |  |
| 5 | ++ | + | + | | ++ | - | | - |  |
| 6 | ++ | + | + | | + | - | | - |  |
| 7 | + | + | + | | ++ | - | | - |  |
| 8 | + | + | + | | + | - | | - |  |
| 9 | ++ | + | ++ | | ++ | + | | - |  |
| 10 | ++ | + | + | | +++ | + | | - |  |
| 11 | ++ | ++ | + | | ++ | + | | - |  |
| 12 | ++ | + | + | | + | - | | - |  |
| 13 | ++ | ++ | + | | ++ | + | | - |  |
| 14 | + | ++ | + | | + | + | | + |  |
| 15 | ++ | ++ | + | | + | + | | - |  |
| 16 | + | ++ | + | | + | - | | - |  |
| 17 | ++ | + | + | | ++ | + | | - |  |
| 18 | ++ | + | + | | ++ | + | | - |  |
| 19 | +++ | ++ | + | | ++ | + | | - |  |
| 20 | ++ | ++ | ++ | | + | - | | - |  |
| Aggregate of + or ++ or +++ | 20/20 | 20/20 | 20/20 | | 20/20 | 11/20 | | 2/20 |  |

PTS, peritumoral stroma; TNs, tumor nests. (+) indicates weak staining, (++) indicates moderate staining, (+++) indicates strong staining, whereas (–) indicates no staining.


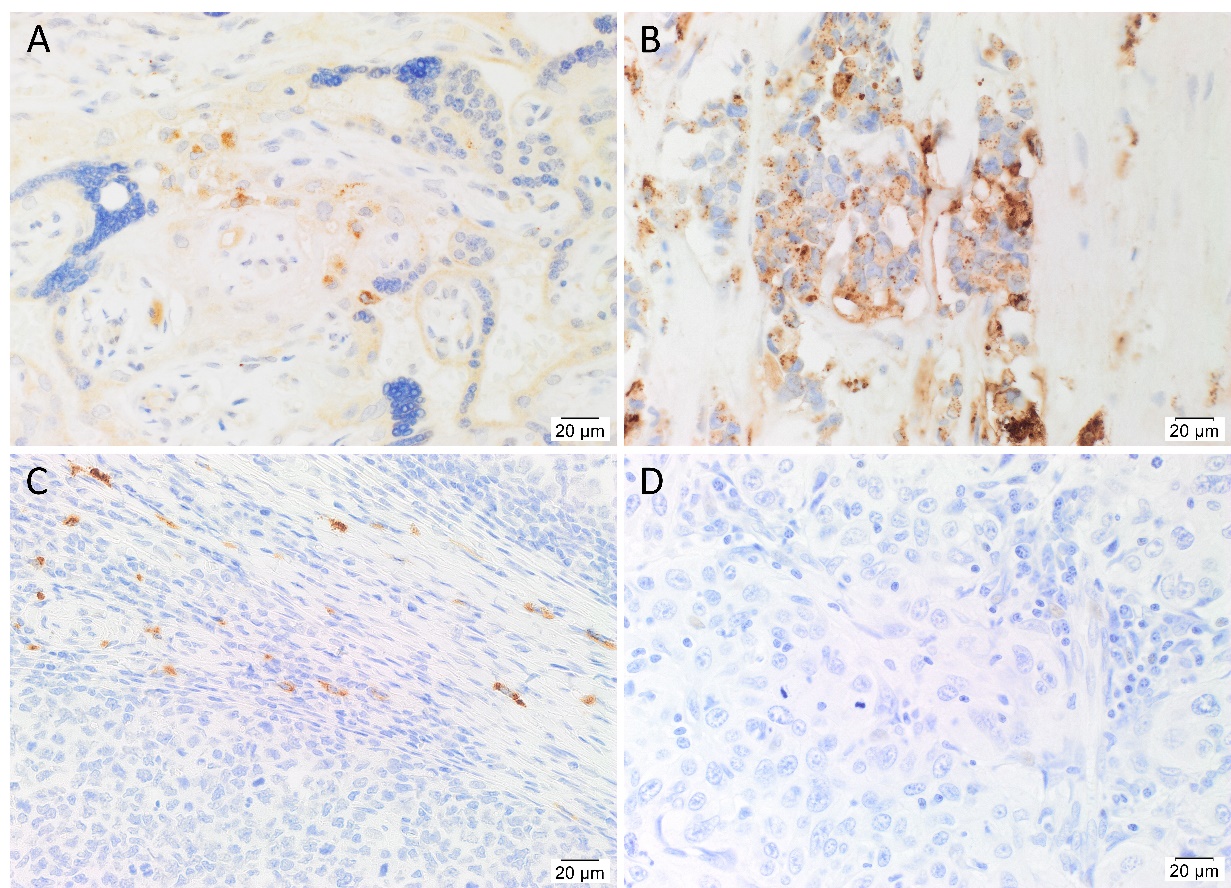
**Supplementary Figure 1.** Human tissues used as positive controls demonstrated the expected positive staining patterns for cathepsin B on placenta (A, brown), cathepsin D on breast carcinoma (B, brown), and cathepsin G (C, brown) on tonsil. A negative matched anti-mouse and anti-rabbit isotype control confirms specificity of the primary antibodies (D, brown). Nuclei were counter-stained with hematoxylin. Nuclei were counter-stained with hematoxylin. Original magnification: 400x. Scale bars: 20µm.


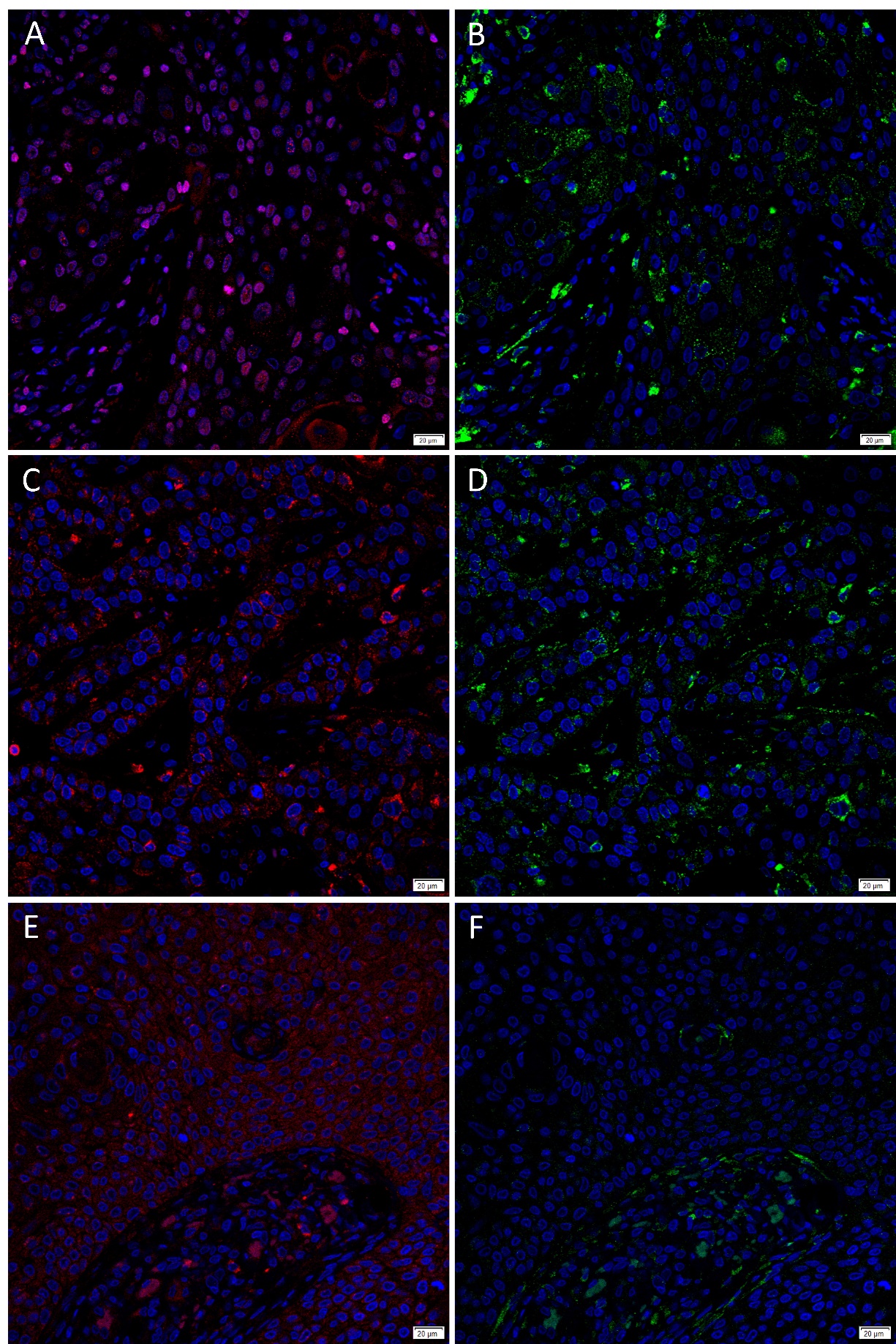


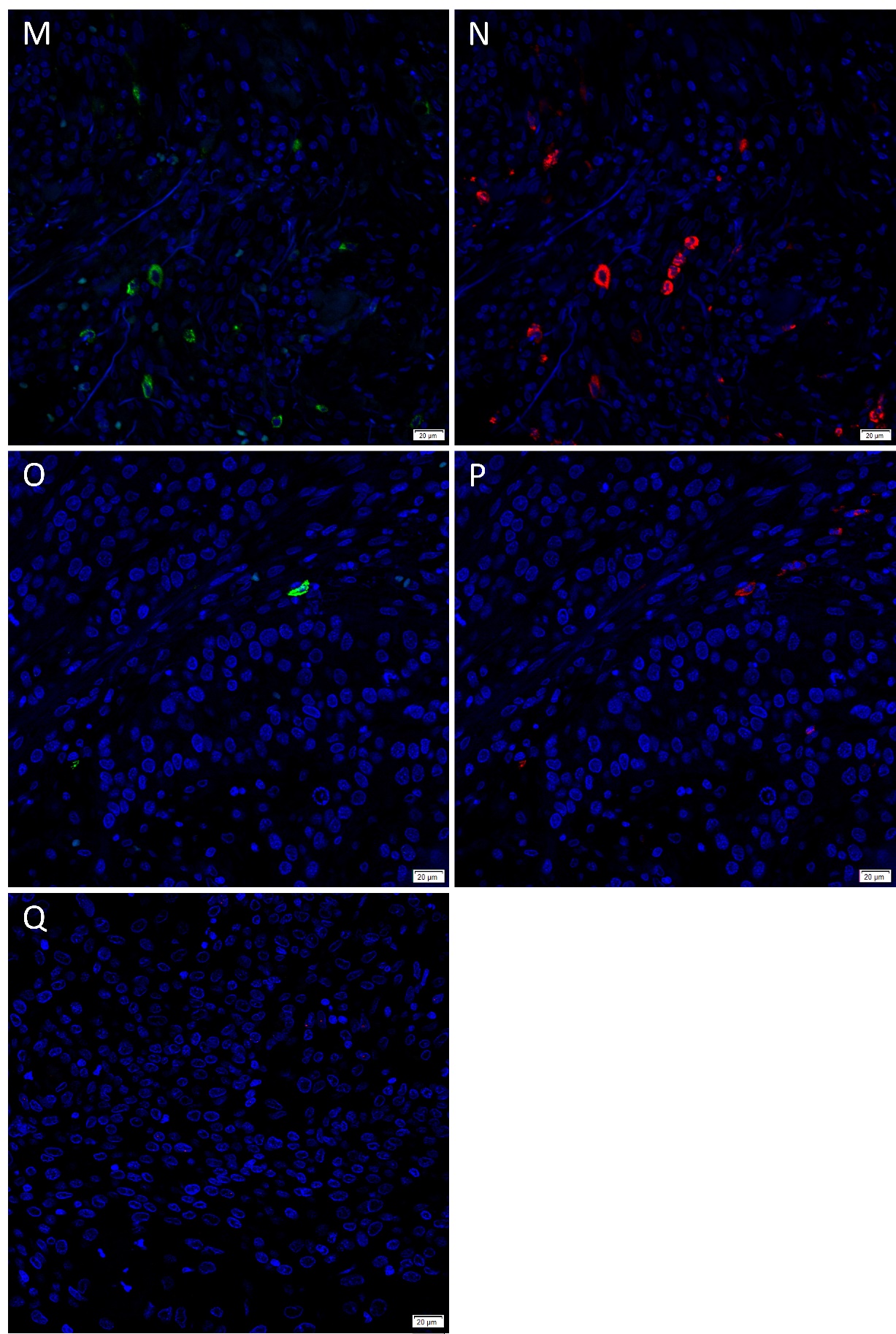
**Supplementary** **Figure 2.** Individual images of immunofluorescence stains of metastatic cutaneous head and neck squamous cell carcinoma (mHNcSCC) sections presented in Figure 2, demonstrating expression of cathepsin B (A, green) and SOX2 (B, red); cathepsin B (C, green) and cathepsin D (D, red); OCT4 (F, green) and cathepsin B (E, red); OCT4 (G, green) and cathepsin D (H, red); tryptase (I, green) and cathepsin G (J, red); chymase (K, green) and cathepsin G (L, red). A negative control (M) confirmed the specificity of the fluorescent secondary antibodies on a section of (mHNcSCC) tissue sample. Cell nuclei were counter-stained with 4’,6-diamidino-2-phenylindole (A-M, blue). Original magnification: 400x. Scale bars: 20µm.


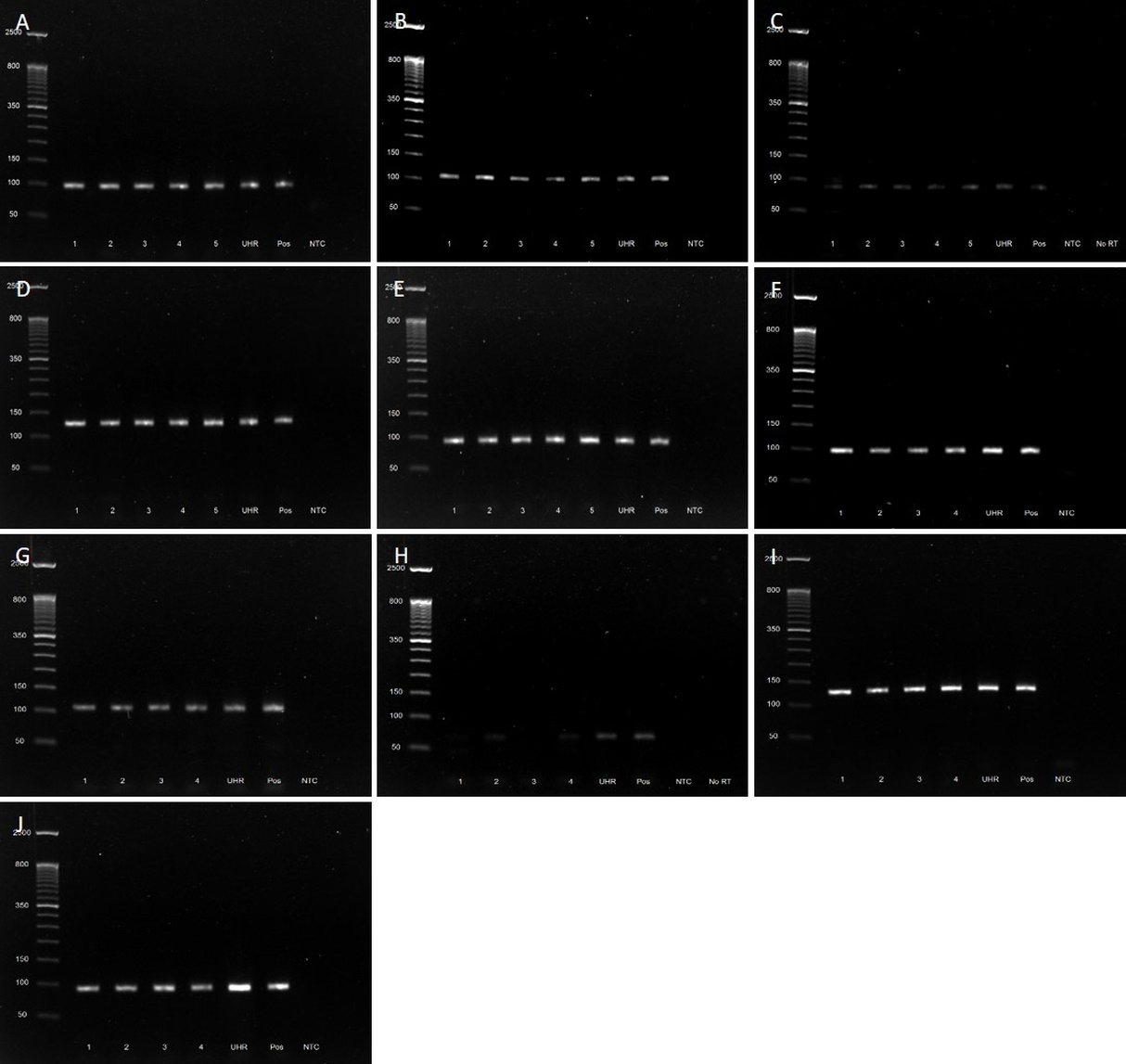


**Supplementary Figure 3.** Reverse transcription quantitative polymerase chain reaction amplification products from five metastatic head and neck cutaneous squamous cell carcinoma (mHNcSCC) tissue samples (A-E) and four mHNcSCC-derived primary cell lines (F-J) were checked using agarose gel electrophoresis. Five mHNcSCC tissue replicate qPCR amplification products were probed for cathepsin B (A, 94bp), cathepsin D (B, 103bp), and cathepsin G (C, 62bp). Four mHNcSCC-derived primary cell lines qPCR amplification products were probed for cathepsin B (G, 94bp), cathepsin D (H, 103bp) and cathepsin G (I, 62bp). GAPDH (122bp) and PUM1 (89bp) were the reference genes from mHNcSCC tissue samples (E) and (F) and cell lines (I) and (J) that were also checked. The ladder refers to the DNA marker in base pairs (bp); Lanes 1-5 refer to the respective tissue/cell samples; Pos, positive control (tonsil tissue for cathepsins B, D and G); NTC, no template control (RNase-free water) to confirm no contamination; No RT, reverse transcriptase negative control for primers that may detect genomic DNA.
